# Stroke Center Certification and Within-Hospital Racial Disparities in Treatment

**DOI:** 10.1001/jamanetworkopen.2025.24027

**Published:** 2025-07-30

**Authors:** Renee Y. Hsia, Yu-Chu Shen

**Affiliations:** 1 Department of Emergency Medicine, University of California, San Francisco; 2 Philip R. Lee Institute for Health Policy Studies, University of California, San Francisco; 3 Department of Defense Management, Naval Postgraduate School, Monterey, California; 4 National Bureau of Economic Research, Cambridge, Massachusetts

## Abstract

**Importance:**

Despite improvements in access to stroke technology, it remains unclear whether Black and White patients with stroke experience similar benefits after a hospital becomes stroke certified and whether stroke center expansion has changed disparities between Black and White patients over time.

**Objective:**

To examine the association of hospital stroke center certification with receipt of acute ischemic stroke treatments and health outcomes between Black and White patients with stroke.

**Design, Setting, and Participants:**

This cohort study implemented a linear probability model with hospital fixed effects to evaluate changes in outcomes for Black and White patients, comparing outcomes before and after a hospital was certified as a stroke center (treatment group) relative to changes in outcomes at hospitals that did not acquire stroke certification (control group). Participants included patients with acute ischemic stroke who were covered by Medicare fee-for-service, who lived in urban communities, and who were admitted to hospitals between January 1, 2009, and December 31, 2019. Data were analyzed from September 1, 2024, to April 30, 2025.

**Exposure:**

Admission to a certified stroke center.

**Main Outcomes and Measures:**

Probability of (1) receipt of thrombolytic therapy, (2) receipt of mechanical thrombectomy, and (3) being home at 90 days and (4) 1-year mortality.

**Results:**

Among 2 109 075 million admissions of patients with stroke included in the analysis, 15.3% were Black, 84.7% were White, 56.8% were female, 15.3% were 65 to 69 years of age, 16.4% were 70 to 74 years of age, 17.7% were 75 to 79 years of age, 18.8% were 80 to 84 years of age, and 31.9% were 85 years or older. Among White patients, the probability of receiving thrombolytic therapy increased by 1.70 (95% CI, 1.19-2.21) percentage points when a hospital became a primary stroke center (PSC) and 3.76 (95% CI, 2.89-4.62) percentage points when a hospital became a thrombectomy-capable or comprehensive stroke center (TSC or CSC), relative to White patients at non–stroke-certified hospitals. Among Black patients, the probability of receiving thrombolytic therapy did not change when admitted to a new PSC or a new TSC or CSC compared with Black patients at non–stroke-certified hospitals. For thrombectomy, a new TSC or CSC was associated with an increase of 3.74 (95% CI, 3.02-4.45) percentage points for White patients and 0.97 (95% CI, 0.03-1.90) for Black patients. No improvements in being home at 90 days or in 1-year mortality were observed.

**Conclusions and Relevance:**

In this cohort study, the likelihood of receiving stroke treatments increased for White but not Black patients within the same facility after the center was stroke certified as a PSC or a TSC or CSC. These within-hospital racial differences serve as sobering evidence that racial disparities in stroke care persist despite increased access to care.

## Key Points

**Question:**

Is stroke center certification associated with improved receipt of stroke treatments and health outcomes equitably for Black and White patients with Medicare who are treated in the same hospital?

**Findings:**

In this cohort study of 2.1 million patient hospitalizations with Medicare fee-for-service from 2009 to 2019, stroke center certification was associated with a significant increase in the use of thrombolytic therapy and thrombectomy for White patients but not for Black patients. No significant improvements were observed for either group in the likelihood of being home at 90 days or 1-year mortality.

**Meaning:**

These findings suggest that racial disparities in stroke care persist despite increased access to care.

## Teaser

This cohort study assesses whether benefits of stroke center certification are equally experienced by Black and White patients with stroke.

## Introduction

Stroke has been called a “disease of disparities,” since differential gradients for historically underserved individuals and those living in areas of disadvantage are present across the entire continuum of the disease.^1^ Even after adjustment for these differences, Black patients with stroke have more than double the risk of death compared with White patients,^2^ and they are one-fifth as likely to receive thrombolytic therapy,^3^ discrepancies that are not explained by contraindications. Although disease incidence,^4^ comorbidities, and other risk factors influence outcomes, there is clear evidence that, for clinically similar patients, substantial disparities in morbidity and mortality are driven by inequalities in access and treatment patterns.^5,6,7,8,9^

The treatment landscape for stroke shifted monumentally in 2015 with landmark publications of randomized clinical trials showing the benefit of endovascular thrombectomy,^10,11,12,13,14^ which was accompanied by a substantial rise in the number of thrombectomies performed over time.^15^ These shifts in the available treatment options for stroke represented a significant opportunity to study how the diffusion of stroke technology has affected racial disparities across the US. Since 2004, hospitals have had the opportunity to seek certification from various organizations to be recognized as stroke centers at different levels, now categorized as acute stroke–ready hospitals (ASRHs), primary stroke centers (PSCs), thrombectomy-capable stroke centers (TSCs), and comprehensive stroke centers (CSCs). The use of telemedicine, or telestroke, for treating patients with acute stroke has grown significantly as an approach to expand access to treatment, particularly for those in rural areas and with public insurance.^16,17,18,19,20^ Receiving care at a certified stroke center has been associated with a reduction in mortality for patients with acute stroke.^21,22,23,24^ There has been a substantial increase in patients with access to stroke technology and admission to certified stroke centers during the past decade.^25^ However, we also know that access to stroke centers is not even across all populations; in other words, more advantaged and wealthier communities have a higher likelihood of hospitals nearby receiving stroke center certification.^26,27^ Additionally, Black racially segregated communities have the lowest likelihood of per-capita access to a stroke-certified hospital when compared with non-Black racially segregated communities.^28^

However, a critical gap in knowledge persists: it is unclear whether the benefits of stroke center certification are equally experienced by Black and White patients with stroke and how this has influenced the disparity between Black and White patients over time. We hypothesized that all else being equal, both Black and White patients with stroke admitted to hospitals with newly acquired stroke center certification would see similar improvements in access to treatment (defined as receipt of thrombolytics and thrombectomy) and health outcomes, ultimately narrowing the disparity gap patients. This information is vital in our efforts to identify mechanisms and effectively target resources or interventions.

## Methods

This cohort study was approved by the Institutional Review Board of the National Bureau of Economic Research, which did not require informed consent for the use of deidentified patient records. We followed the Strengthening the Reporting of Observational Studies in Epidemiology (STROBE) reporting guidelines.

### Data Sources and Study Population

We obtained patient data from the 100% Medicare Provider and Analysis Review (MedPAR) files between 2009 and 2019, with relevant data elements of date and site of admission, diagnosis and procedure codes, and the patients’ mailing zip codes. We combined the MedPAR data with Medicare Beneficiary Summary Files to obtain demographic information (age, sex, race and ethnicity, and eligibility for Medicaid) and date of death (if applicable) to construct mortality outcomes. We further merged these patient data with data from the US Census and the Federal Office of Rural Health Policy^29^ to identify geographic coordinates and whether a patient’s community (proxied by zip code) should be classified as rural or urban.

Our hospital data were derived from several different sources. First, we collected data on stroke center certification status from national accrediting bodies as well as from states.^30^ Second, we captured additional hospital geographic information (ie, longitude and latitude) and operating characteristics from the American Hospital Association and the Healthcare Cost Report Information System.

Our patient population included 100% Medicare fee-for-service patients from urban communities whose principal diagnosis was acute ischemic stroke and who were admitted to hospitals between January 1, 2009, and December 31, 2019. We identified this patient population based on *International Classification of Diseases, Ninth Revision* (*ICD-9*), codes 433.x1, 434.x1, or 436, effective until September 30, 2015, or *International Statistical Classification of Diseases and Related Health Problems, Tenth Revision* (*ICD-10*), code I63, effective on and after October 1, 2015, in accordance with prior literature.^31,32,33^ We excluded rural communities because patients from rural communities face an entirely different set of challenges with the availability of stroke resources^34^ and warrant a separate analysis and discussion. We also excluded patients whose communities were more than 100 miles away from the admitting hospital because they were most likely admitted while away from home. Since our analysis focused on investigating disparities between Black and White patients with stroke within hospitals, we only included patients who self-identified as either Black or White based on the Medicare Beneficiary Summary Files.

### Identifying Stroke Center Certification Levels and Date of Certification

We collected hospital stroke center certification information from national accrediting organizations, including the Joint Commission, Det Norske Veritas, Accreditation Commission for Health Care, and Center for Improvement in Healthcare Quality, in addition to state departments of health.^27,30^ Following prior studies,^34^ we grouped stroke certification levels into the following mutually exclusive categories (from least to most advanced): ASRH, PSC, TSC, and CSC. ASRHs are more common in rural communities because their main purpose is to rapidly assess and stabilize patients with stroke, which can include the administration of thrombolytic therapy and having protocols in place to transfer patients to higher-level stroke centers. PSCs were the first type of stroke certification to be established with dedicated stroke resources, including diagnosis, treatment, and monitoring for acute strokes, with medical personnel specialized in treating strokes. The most advanced stroke centers are TSCs and CSCs, which are certified to perform mechanical thrombectomy and have established volume criteria for certification. CSCs are required to have additional resources, such as 24-hour neurointensivist coverage.

Due to the low number of hospitals receiving TSC designation, we combined TSC and CSC into 1 certified group. The PSC indicator became 1 on and after the quarter the hospital became PSC certified and otherwise remained as 0. If a PSC hospital became a CSC or TSC in a later quarter, the CSC or TSC indicator changed to 1 on and after that quarter, while the PSC indicator turned back to 0.

### Outcomes

To determine whether Black and White patients with stroke experienced similar outcomes after being admitted to a hospital that acquired stroke center certification, we evaluated both treatment and health outcomes. We focused on 2 treatment outcomes: (1) receipt of thrombolysis and (2) receipt of mechanical thrombectomy using *ICD-9* and *ICD-10* procedure codes as in previous literature.^35,36,37^ Both treatments are part of the accepted clinical guidelines for the early treatment of acute stroke,^38^ as they have been demonstrated to improve functional independence.^10,11,12,39,40,41^ For health outcomes, we analyzed 2 metrics: (1) home at 90 days (eg, not in an inpatient acute or rehabilitation hospital) and (2) mortality within 1 year of admission (1-year mortality).

### Statistical Analysis

Using a difference-in-differences framework for our patient-level analysis, we implemented linear probability models with hospital fixed effects to compare changes in patient outcome probabilities before and after a hospital was certified at a given stroke care level (treatment group), relative to changes in hospitals that did not acquire stroke center certification (control group), during the same period. Although a probit or logit model might be considered a more natural choice for estimating a dichotomous variable in cross-sectional data, these models would result in inconsistent estimators in panel data because we included a large number of hospital fixed effects.^42^ These hospital fixed effects were critical for our identification because they removed unobserved differences across hospitals (such as underlying socioeconomic status and case severity). We estimated the coefficients separately for Black and White patients. For each race, the 3 key variables—indicators for certification as ASRH, PSC, or TSC or CSC—took on a value 1 beginning in the quarter when the admitting hospital achieved that certification level, and 0 otherwise. We included time dummies to control for secular trends, as well as patient sociodemographic covariates (sex, 5-year age groups, and Medicaid eligibility) and disease-related risk adjustments, following prior work.^43,44^ In our main model, we included patients from all hospitals as defined in our study population above. In our sensitivity analysis, we limited the sample to patients admitted to hospitals that treated both Black and White patients across all years. Additional model details are included in the eMethods in Supplement 1.

For our exploratory analysis examining changes in patient volumes and patient profiles after a hospital became a certified stroke center, we conducted a hospital-level analysis where each observation represented a hospital-year quarter. We focused on the following hospital-level patient profile measures: changes in patient volume (log-transformed due to skewed distributions and for ease of interpreting coefficients), mean age of admitted patients with stroke, mean number of comorbid conditions, and percentage of cases that were recurring strokes. For each patient profile measure, we also studied mean differences within hospitals between Black and White patients with stroke. Using the same difference-in-differences framework as our main analysis, we implemented an ordinary least squares regression with hospital fixed effects to control for underlying differences in hospital culture and coding practices, as well as time dummies to control for macro trends in patient volumes and overall changes in age and comorbidity counts, regardless of certification status. All analyses were completed using Stata, version 18 (StataCorp LLC) between September 1, 2024, to April 30, 2025. Two-sided *P* < .05 (Bonferroni adjusted for multiple tests) indicated statistical significance.

## Results

**Figure 1.**
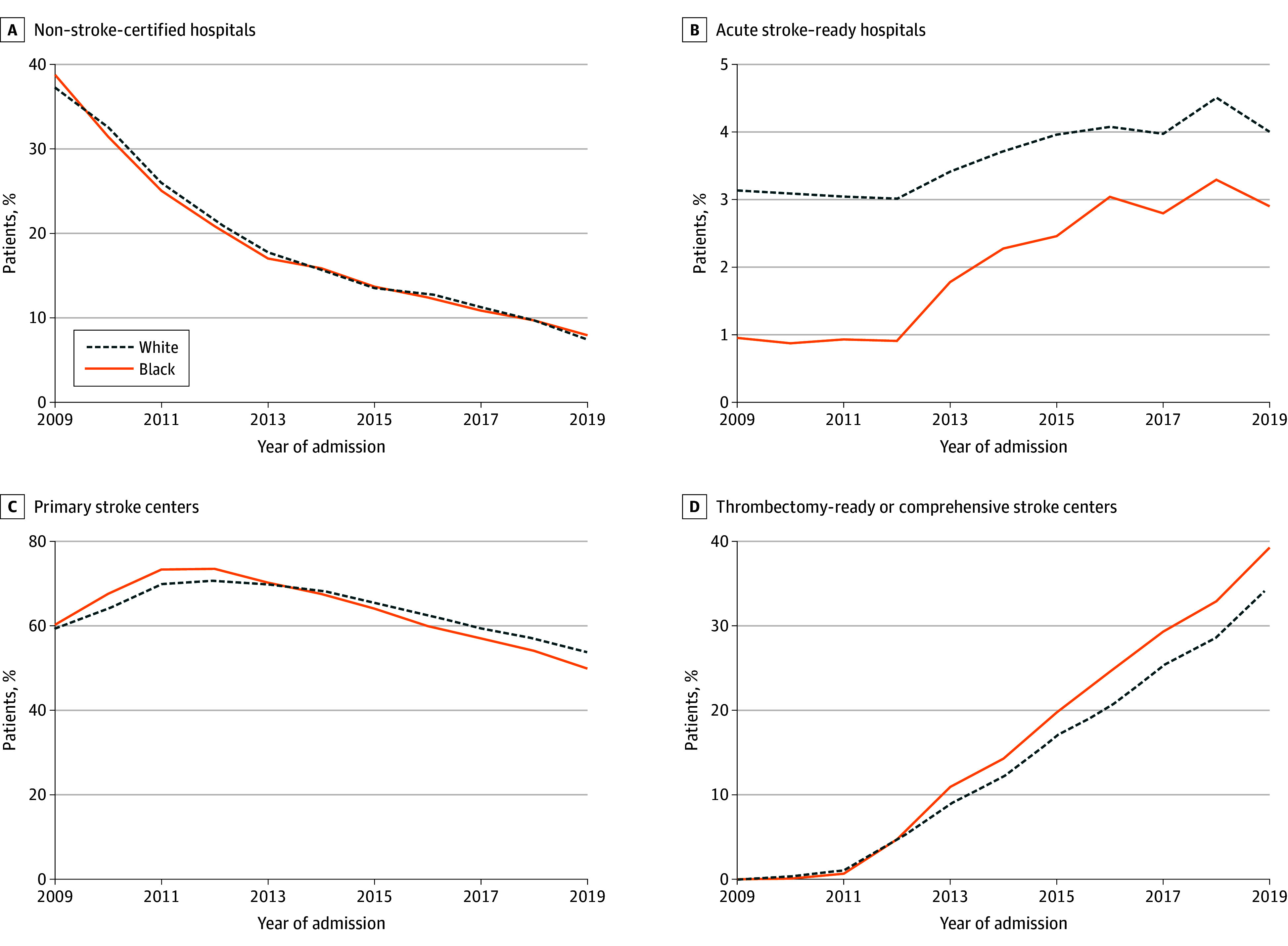
Time Patterns in Percentage of Patients Admitted to Hospitals by Stroke Center Certification Level and Patient Race Includes 323 292 Black and 1 785 783 White patients admitted from January 1, 2009, to December 31, 2019.

Our final study population contained 2 109 075 admissions of patients with stroke during the study period across 3210 hospitals. Of these patients, 323 292 (15.3%) were Black and 1 785 783 (84.7%) were White; 1 197 112 (56.8%) were female and 911 963 (43.2%) were male; 321 872 (15.3%) were 65 to 69 years of age, 346 089 (16.4%) were 70 to 74 years of age, 372 642 (17.7%) were 75 to 79 years of age, 396 727 (18.8%) were 80 to 84 years of age, and 671 754 (31.9%) were 85 years or older. Figure 1 reveals patterns in the percentage of Black and White patients with stroke in urban communities who were admitted to each type of stroke hospital between 2009 and 2019. For both races, the percentage of patients admitted to non–stroke-certified hospitals decreased from 38.8% in 2009 to less than 8% in 2019 (Figure 1A). About 60% of patients from both races were admitted to a PSC in 2009; by 2019, 49.9% of Black and 53.9% of White patients were admitted to a PSC (Figure 1C). There was a dramatic increase in the share of patients admitted to a TSC or CSC from 0 in 2009 (since the higher-level certification did not exist until 2012) to 39.3% of Black patients and 34.7% of White patients in 2019 (Figure 1D).

Table 1 shows patient characteristics separately by the hospital’s highest level of stroke certification (ie, if a PSC became a TSC during the study period, all patients admitted to that hospital would be grouped into the TSC category). Table 1 shows that White patients represented 91.1% of all patients in hospitals with no stroke certification but only 82.0% in hospitals that achieved a TSC or CSC level. On the other hand, Black patients with stroke represented 8.9% of all patients in hospitals with no stroke certification but 18.0% in TSCs or CSCs. The age distribution was similar across certification levels, with the exception that patients in TSCs or CSCs were slightly older (32.8% were 85 years or older compared with 30.1% among patients in non–stroke-certified hospitals). The share of patients with each comorbidity was also similar across all certification levels. In comparing Black and White patients regardless of where they were admitted, eTable 1 in Supplement 1 shows that Black patients tended to be younger (25.4% were in the group aged 65-69 years compared with 13.4% among White patients) and had a higher likelihood of being Medicaid eligible (27.8% compared with 9.5% among White patients). The distribution of comorbid conditions also differed between Black and White patients. Our models controlled for these individual differences.

**Table 1.** Descriptive Statistics of Patient Characteristics, by Stroke Center Certification Level of Admitted Hospital

Characteristic	Hospital’s highest level of stroke center certification achieved during study period, No. (%)			
	No certification (n = 250 281)	ASRH (n = 48 435)	PSC (n = 395 242)	TSC or CSC (n = 1 415 117)
Race				
Black	22 232 (8.9)	5417 (11.2)	40 759 (10.3)	254 884 (18.0)
White	228 049 (91.1)	43 018 (88.8)	354 483 (89.7)	1 160 233 (82.0)
Sex				
Female	137 330 (54.9)	26 807 (55.3)	222 032 (56.2)	810 943 (57.3)
Male	112 951 (45.1)	21 628 (44.7)	173 210 (43.8)	604 174 (42.7)
Age, y				
65-69	38 652 (15.4)	8354 (17.2)	61 764 (15.6)	213 102 (15.1)
70-74	43 013 (17.2)	8717 (18.0)	67 279 (17.0)	227 080 (16.0)
75-79	45 975 (18.4)	8909 (18.4)	72 135 (18.3)	245 623 (17.4)
80-84	47 397 (18.9)	9049 (18.7)	75 041 (19.0)	265 240 (18.7)
≥85	75 246 (30.1)	13 406 (27.7)	119 023 (30.1)	464 079 (32.8)
Eligible for Medicaid	29 422 (11.8)	6788 (14.0)	45 435 (11.5)	177 391 (12.5)
Patient clinical conditions				
Recurring stroke	21 227 (8.5)	4055 (8.4)	34 082 (8.6)	126 018 (8.9)
Transfer	15 677 (6.3)	4584 (9.5)	25 473 (6.4)	57 662 (4.1)
Peripheral vascular disease	24 187 (9.7)	4674 (9.7)	39 281 (9.9)	145 068 (10.3)
Pulmonary circulation disorders	9304 (3.7)	1376 (2.8)	13 901 (3.5)	51 155 (3.6)
Diabetes	79 108 (31.6)	16 037 (33.1)	127 275 (32.2)	451 324 (31.9)
Kidney failure	44 594 (17.8)	8818 (18.2)	73 964 (18.7)	271 909 (19.2)
Liver	2309 (0.9)	430 (0.9)	3828 (1.0)	14 338 (1.0)
Cancer	9867 (3.9)	1810 (3.7)	15 462 (3.9)	59 107 (4.2)
Dementia	25 712 (10.3)	4898 (10.1)	41 926 (10.6)	157 157 (11.1)
Valvular disease	25 662 (10.3)	4219 (8.7)	38 773 (9.8)	140 537 (9.9)
Hypertension	210 336 (84.0)	41 014 (84.7)	334 420 (84.6)	1 205 383 (85.2)
Chronic pulmonary disease	42 668 (17.0)	8063 (16.6)	66 641 (16.9)	217 860 (15.4)
Rheumatoid arthritis or collagen vascular disease	7142 (2.9)	1373 (2.8)	11 414 (2.9)	39 264 (2.8)
Coagulation deficiency	9516 (3.8)	1564 (3.2)	13 998 (3.5)	55 405 (3.9)
Obesity	20 779 (8.3)	4092 (8.4)	33 614 (8.5)	115 630 (8.2)
Substance use	5812 (2.3)	1054 (2.2)	8397 (2.1)	32 036 (2.3)
Depression	24 641 (9.8)	4762 (9.8)	40 218 (10.2)	136 110 (9.6)
Psychosis	16 362 (6.5)	3341 (6.9)	27 313 (6.9)	98 136 (6.9)
Hypothyroidism	44 847 (17.9)	8397 (17.3)	73 489 (18.6)	244 664 (17.3)
Paralysis and other neurological disorders	142 164 (56.8)	28 187 (58.2)	225 069 (56.9)	814 100 (57.5)
Ulcer	807 (0.3)	174 (0.4)	1236 (0.3)	4823 (0.3)
Weight loss	9626 (3.8)	2068 (4.3)	16 588 (4.2)	65 433 (4.6)
Fluid and electrolyte disorders	56 918 (22.7)	10 694 (22.1)	89 397 (22.6)	327 020 (23.1)
Anemia (blood loss and deficiency)	29 924 (12.0)	5594 (11.5)	47 542 (12.0)	185 418 (13.1)
Access, treatment received, and health outcomes				
Thrombolytic therapy	23 462 (9.4)	4783 (9.9)	36 601 (9.3)	148 671 (10.5)
Thrombectomy	5504 (2.2)	1117 (2.3)	8474 (2.1)	41 510 (2.9)
Outcome				
Home at 90 d	173 578 (69.4)	33 127 (68.4)	273 961 (69.3)	985 414 (69.6)
1-y Mortality	70 939 (28.3)	13 853 (28.6)	112 342 (28.4)	401 232 (28.4)

Abbreviations: ASRH, acute stroke–ready hospital; CSC, comprehensive stroke center; PSC, primary stroke center; TSC, thrombectomy-capable stroke center.

**Figure 2.**
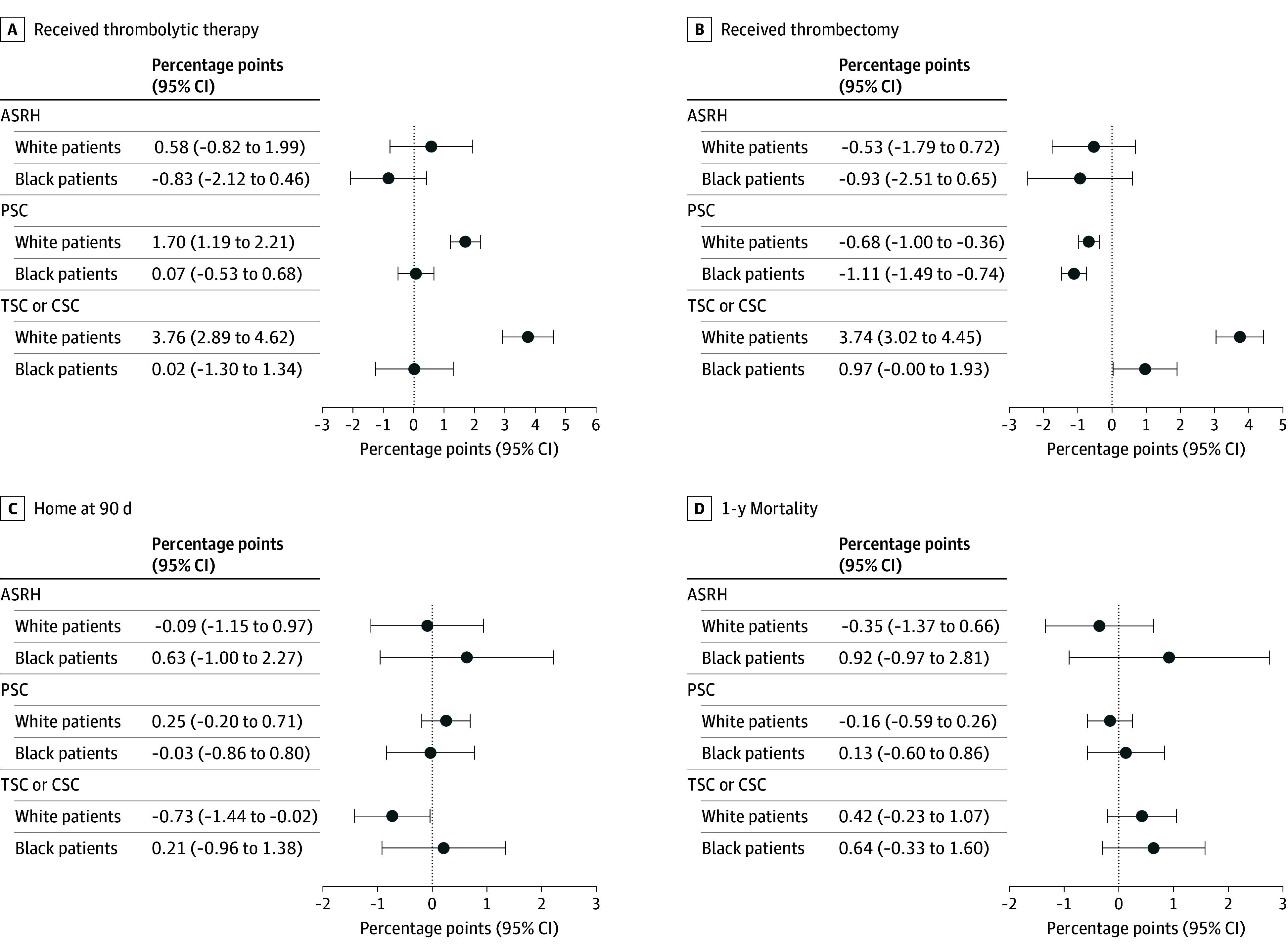
Changes in Probabilities of Treatment and Health Outcomes for Black and White Patients With Stroke When Their Admission Hospital Gained Stroke Certification Includes 323 292 Black and 1 785 783 White patients admitted from January 1, 2009, to December 31, 2019. Error bars represent Bonferroni-adjusted 95% CIs to account for multiple testing. ASRH indicates acute stroke–ready hospital; CSC, comprehensive stroke center; PSC, primary stroke center; and TSC, thrombectomy-capable stroke center.

Figure 2 illustrates the main results from our entire patient sample (full results in eTable 2 in Supplement 1). Figure 2A shows that for White patients, the probability of receiving thrombolytic therapy improved by 1.70 (Bonferroni-adjusted 95% CI for multiple testing, 1.19-2.21) percentage points when a hospital became a PSC and 3.76 (95% CI, 2.89-4.62) when a hospital became a TSC or CSC, relative to White patients who were admitted to non–stroke-certified hospitals. Given that the mean rate of thrombolytic therapy was 5.6% for White patients in 2009, this is equivalent to a 30.5% increase when hospitals became certified as PSCs and a 67.7% increase when hospitals became certified as TSCs or CSCs. On the other hand, the probability of thrombolytic therapy did not change for Black patients admitted to hospitals that received certification as a PSC or a TSC or CSC, compared with their Black counterparts in hospitals that did not gain certification. The changes in receipt of thrombolytic therapy were not statistically significant for both Black and White patients with stroke who were admitted to ASRHs. The probability of thrombectomy decreased after a hospital became a PSC, by 0.68 (95% CI, −1.00 to −0.36) percentage points for White patients and 1.11 (95% CI, −1.40 to −0.82) percentage points for Black patients, but soared for White patients after a hospital became a TSC or CSC (3.74 [95% CI, 3.02-4.45] percentage points; equivalent to a 506% relative increase given the mean baseline rate of 0.74%) and increased moderately for Black patients (0.97 [95% CI, 0.03-1.90] percentage points; equivalent to a 137% relative increase given the mean baseline rate of 0.69%). White patients saw a decrease of 0.73 (95% CI, −1.44 to −0.02) percentage points in home at 90 days but no change in 1-year mortality after a hospital became a TSC or CSC. We did not observe any significant changes in home at 90 days or 1-year mortality for Black patients when hospitals acquired stroke certification at any level.

In our hospital-level analysis, we investigated whether the changes in treatment and outcomes observed in Figure 2 might be driven by changes in patient volumes and patient profiles. Table 2 shows that the overall patient volume rose by 15.4% (95% CI, 11.6%-19.1%) after a hospital became a PSC relative to a hospital that did not acquire stroke certification and by 39.2% (95% CI, 33.8%-44.7%) when a hospital became a TSC or CSC. In the whole sample, the proportion of Black patients did not change significantly. The mean patient age also did not change significantly, but the admitted patient population had a higher number of comorbidities; specifically, the mean number of comorbidities grew by 0.14 (95% CI, −0.03 to 0.30) after the hospital became a PSC and by 1.00 (95% CI, 0.73-1.28) when a hospital became a TSC or CSC. Still, there was no statistically significant difference in comorbidity changes between Black and White patients.

**Table 2.** Changes in Patient Volumes and Patient Profiles After Gaining Stroke Certification^a^

Certification	Overall^b^				Share of Black patients with stroke (95% CI), percentage points	Difference between Black and White patients^c^		
	Log-transformed coefficient patient volume (95% CI), %	Mean age (95% CI), y	Mean comorbidity counts (95% CI)	Share of cases with chronic conditions (95% CI), percentage points		Mean age (95% CI), y	Mean comorbidity counts (95% CI)	Share of cases with chronic conditions (95% CI), percentage points
ASRH	−2.1 (−10.2 to 6.1)	−0.03 (−0.46 to 0.40)	−0.23 (−0.63 to 0.16)	−0.46 (−1.88 to 0.96)	0.69 (−0.81 to 2.19)	−0.23 (−1.09 to 0.63)	0.07 (−0.39 to 0.52)	−0.01 (−0.03 to 0.01)
PSC	15.4 (11.6 to 19.1)^d^	−0.04 (−0.17 to 0.10)	0.14 (−0.03 to 0.30)^e^	−0.35 (−0.88 to 0.18)	−0.14 (−0.678 to 0.39)	−0.01 (−0.35 to 0.34)	−0.02 (−0.21 to 0.17)	−0.00 (−0.01 to 0.01)
TSC or CSC	39.2 (33.8 to 44.7)^d^	0.04 (−0.16 to 0.24)	1.00 (0.73 to 1.28)^d^	0.79 (0.06 to 1.52)^d^	−0.45 (−1.32 to 0.43)	0.13 (−0.36 to 0.62)	0.04 (−0.23 to 0.31)	−0.01 (−0.02 to 0.00)^e^

Abbreviations: ASRH, acute stroke–ready hospital; CSC, comprehensive stroke center; PSC, primary stroke center; TSC, thrombectomy-capable stroke center.

^a^ *P* values and 95% CIs are Bonferroni adjusted to account for multiple testing. Reference group consists of hospitals with no stroke center certification.

^b^ Includes 100 945 observations and 3210 hospitals.

^c^ Includes 54 784 observations and 2328 hospitals.

^d^ *P* < .01.

^e^ *P* < .05.

In our sensitivity analysis, we restricted our patient sample to only those admitted to hospitals that treat both Black and White patients in all years, and our results remained similar to the main model (eFigure in Supplement 1). The estimated coefficients in our main model are likely conservative because some hospitals may begin enhancing their stroke care capacity 12 to 24 months prior to the certification date to fulfill the certification requirements. To investigate this possibility, we performed a sensitivity analysis that expanded our main model to include 3 different periods: the precertification period (reference period), the planning period (12-24 months preceding the certification date), and the postcertification period (on and after a hospital was certified). For White patients with stroke, we observed a ramp-up of both thrombolytic and thrombectomy treatments, especially in TSCs or CSCs (eTable 3 in Supplement 1). For Black patients, there was no significant change in thrombolytic therapy across all certification levels and during both time periods. We detected a ramp-up of thrombectomy with Black patients admitted to TSCs or CSCs, although the magnitude of this change was substantially smaller than for White patients.

## Discussion

Our cohort study of more than 2 million Medicare beneficiaries with acute ischemic stroke in the US showed that the likelihood of receiving stroke treatment increased for White patients but not Black patients after a hospital became stroke certified at the PSC level or higher, even when comparing patients within the same hospital. Specifically, the likelihood of receiving thrombolytic therapy increased by 31% after a PSC certification and by 68% after a TSC or CSC certification for White patients with stroke who were admitted to hospitals that became stroke-certified compared with White patients whose hospitals were not stroke certified. However, there were no statistically significant changes in the probability of thrombolytic therapy when comparing Black patients between the same set of treatment and control hospitals. When examining the likelihood of receiving thrombectomy, both Black and White patients with stroke benefited from hospital certification as a TSC or CSC, but by vastly different amounts: White patients with a 506% increase, and Black patients with a 137% increase. The smaller improvement observed in Black patients is not explained by a higher baseline treatment rate; Black patients were less likely than White patients to receive either treatment at baseline. Thus, our findings provide sobering evidence that the Black-White gap in treatment has widened, rather than contracted, during the past decade, despite greater access to stroke technology and resources.

Given that our results were based on a within-hospital comparison, the observed differences in Black-White treatment rates were not driven by differential access to different types of hospitals. These Black-White differentials were also not driven by differences in patient age or comorbidity profiles, given that in our exploratory analysis, changes in age and comorbidity profiles between Black and White patients were similar before and after hospitals acquired stroke center certifications. We also controlled for these factors in the main model. Given that Black patients had a lower rate of receiving treatments at baseline in 2009, how are we to understand the disheartening results that the disparity in treatment between Black and White patients with stroke is growing rather than shrinking?

Prior work has shown that Black patients with stroke have lower use of emergency medical services and ambulances than non-Hispanic White patients.^45,46,47^ Other work has shown that Black patients are more likely to have delayed presentation from the last time the patient was reported to be in a normal state (last seen normal) to arrival at the emergency department (ED),^48,49^ leading to lower rates of treatment. Even when they do arrive at the ED, Black patients with stroke are twice as likely to have to wait more than 10 minutes in the ED compared with White patients with stroke.^50^ Studies have also documented a higher rate of refusal of treatment from Black patients with stroke compared with their White counterparts.^51^ Even if these differences have not changed over time, if more patients—both Black and White—are now admitted to certified stroke centers, these disparities could be growing. Our findings suggest that perhaps targeted education on the need for timely recognition and treatment of stroke symptoms in certain populations may be critical in reducing disparities for this condition.

Furthermore, our exploratory analysis showed that patient volumes increased substantially when hospitals acquired higher-level stroke certification, and hospitals received patients with more underlying comorbidities when becoming TSCs or CSCs. It is possible that this surge in patient volumes, as a result of differential certification, correlated with the aforementioned patterns of emergency medical services arrival, ED wait times, and refusal rates between Black and White patients with stroke, which subsequently affected the treatments received. Our data do not have information to distinguish these potential mechanisms, but this would be important for future studies. While the Affordable Care Act was implemented during our study period and may have influenced overall access to care through Medicaid expansion and insurance reforms, stroke center certification is primarily guided by professional society standards rather than Affordable Care Act mandates. Our findings suggest that within-hospital racial disparities in treatment persisted despite broader coverage reforms, indicating that expanded insurance alone may be insufficient to eliminate inequities in stroke care delivery.

### Limitations

This study has several important limitations. First, we used administrative data, which do not include the granularity of clinical detail that would ideally be available. Specifically, we were unable to look at the severity of presenting cases with measures such as the National Institutes of Health Stroke Scale or modified Rankin Scale, nor do we have clinical data regarding the time of onset or last seen normal that would influence a clinician’s decision to treat. As a result, we cannot rule out the possibility that Black patients might be experiencing a smaller relative benefit in treatment due to differences in clinical presentations that are potentially less severe and do not warrant thrombolytics or thrombectomy or have more contraindications to receive tissue plasminogen activator therapy,^52^ as shown in some studies. However, this would only explain the differences if the clinical severity and timing of presentation were changing differentially for Black compared with White patients with stroke over time, which is unlikely. Second, our administrative data also lacked functional outcomes, and we did not find any differences in home at 90 days or 1-year mortality. This is not unexpected, since several observational studies have found lower mortality with thrombolytic therapy,^53,54^ and clinical trials have generally not been able to demonstrate a mortality benefit from receipt of thrombolytics^55^ or thrombectomy. Literature demonstrating more functional independence at 90 days does exist,^10,11,12,39,40,41^ but this is likely less reflected in the home at 90 days measure, which is less precise. Third, while our data contained comorbid conditions so that we could compare clinically similar Black and White patients, we did not have a direct measure of socioeconomic characteristics such as income and educational levels. While we used Medicaid eligibility as a proxy in our model and the hospital fixed effects control for baseline differences in patient socioeconomic status across hospitals, we were not able to ascertain whether the observed differences were driven by changes in these socioeconomic factors. Fourth, we excluded patients in rural communities, so our results were not generalizable to rural populations where stroke care dynamics differ significantly. Last, our study was limited to Medicare fee-for-service patients. However, we did not expect that this would affect our ability to detect any differential effects of stroke center certification between Black and White patients unless patients from these populations systematically differed from privately insured populations or Medicare Advantage populations after stroke center certification.

## Conclusions

Overall, our cohort study of 2.1 million patients with stroke found that White patients with stroke experienced greater benefits in receipt of stroke treatments than Black patients when their admitted hospital was recently certified as a stroke center, even when comparing Black and White patients treated at the same hospitals. Although Black patients experienced an increase in the likelihood of receiving thrombectomy after their hospital was certified, this improvement was significantly smaller than for White patients admitted to the same hospital, and only White patients experienced an increase in the likelihood of receiving thrombolytic therapy. These within-hospital differences between Black and White patients with stroke serve as sobering evidence that significant barriers persist in reducing racial disparities in treatment, despite greater access to stroke technology and resources.

## Notes

Supplement 1. **eMethods.** Additional Details on Statistical Models **eTable 1.** Patient Demographic and Comorbid Characteristics, by Race **eTable 2.** Complete Regression Results of Figure 2 **eTable 3.** Changes in Outcomes During the 12- to 24-Month Precertification Period and the Postcertification Period **eFigure.** Changes in Probabilities of Treatment and Health Outcomes for Black and White Patients With Stroke When Their Admitted Hospital Gains Stroke Certification, Restricted to Hospitals That Treat Patients of Both Races for All Years of the Study Period
eReferences.

Supplement 2.
Data Sharing Statement

## References

[1] Skolarus LE, Sharrief A, Gardener H, Jenkins C, Boden-Albala B. Considerations in addressing social determinants of health to reduce racial/ethnic disparities in stroke outcomes in the United States. Stroke. 2020;51(11):3433-3439. doi:10.1161/STROKEAHA.120.030426 33104471 PMC7732185

[2] Flynn A, Vaughan AS, Casper M. Differences in geographic patterns of absolute and relative Black-White disparities in stroke mortality in the United States. Prev Chronic Dis. 2022;19:E63. doi:10.5888/pcd19.220081 36201790 PMC9541688

[3] Johnston SC, Fung LH, Gillum LA, . Utilization of intravenous tissue-type plasminogen activator for ischemic stroke at academic medical centers: the influence of ethnicity. Stroke. 2001;32(5):1061-1068. doi:10.1161/01.STR.32.5.1061 11340210

[4] Howard G, Moy CS, Howard VJ, ; REGARDS Investigators. Where to focus efforts to reduce the Black-White disparity in stroke mortality: incidence versus case fatality? Stroke. 2016;47(7):1893-1898. doi:10.1161/STROKEAHA.115.012631 27256672 PMC4927373

[5] Schwamm LH, Reeves MJ, Pan W, . Race/ethnicity, quality of care, and outcomes in ischemic stroke. Circulation. 2010;121(13):1492-1501. doi:10.1161/CIRCULATIONAHA.109.881490 20308617

[6] Nasr DM, Brinjikji W, Cloft HJ, Rabinstein AA. Racial and ethnic disparities in the use of intravenous recombinant tissue plasminogen activator and outcomes for acute ischemic stroke. J Stroke Cerebrovasc Dis. 2013;22(2):154-160. doi:10.1016/j.jstrokecerebrovasdis.2011.07.003 22155116

[7] Aparicio HJ, Carr BG, Kasner SE, . Racial disparities in intravenous recombinant tissue plasminogen activator use persist at primary stroke centers. J Am Heart Assoc. 2015;4(10):e001877. doi:10.1161/JAHA.115.001877 26467999 PMC4845141

[8] Lukachko A, Hatzenbuehler ML, Keyes KM. Structural racism and myocardial infarction in the United States. Soc Sci Med. 2014;103(103):42-50. doi:10.1016/j.socscimed.2013.07.021 24507909 PMC4133127

[9] Krieger N. Discrimination and health inequities. In: Berkman LF, Kawachi I, Glymour M, eds. Social Epidemiology. 2nd ed. Oxford University Press; 2014.

[10] Berkhemer OA, Fransen PSS, Beumer D, ; MR CLEAN Investigators. A randomized trial of intraarterial treatment for acute ischemic stroke. N Engl J Med. 2015;372(1):11-20. doi:10.1056/NEJMoa1411587 25517348

[11] Jovin TG, Chamorro A, Cobo E, ; REVASCAT Trial Investigators. Thrombectomy within 8 hours after symptom onset in ischemic stroke. N Engl J Med. 2015;372(24):2296-2306. doi:10.1056/NEJMoa1503780 25882510

[12] Campbell BCV, Mitchell PJ, Kleinig TJ, ; EXTEND-IA Investigators. Endovascular therapy for ischemic stroke with perfusion-imaging selection. N Engl J Med. 2015;372(11):1009-1018. doi:10.1056/NEJMoa1414792 25671797

[13] Goyal M, Demchuk AM, Menon BK, ; ESCAPE Trial Investigators. Randomized assessment of rapid endovascular treatment of ischemic stroke. N Engl J Med. 2015;372(11):1019-1030. doi:10.1056/NEJMoa1414905 25671798

[14] Goyal M, Menon BK, van Zwam WH, ; HERMES Collaborators. Endovascular thrombectomy after large-vessel ischaemic stroke: a meta-analysis of individual patient data from five randomised trials. Lancet. 2016;387(10029):1723-1731. doi:10.1016/S0140-6736(16)00163-X 26898852

[15] Patel K, Hamedani AG, Taneja K, . Differential thrombectomy utilization across hospital classifications in the United States. J Stroke Cerebrovasc Dis. 2023;32(12):107401. doi:10.1016/j.jstrokecerebrovasdis.2023.107401 37897885

[16] Adams HP Jr, del Zoppo G, Alberts MJ, ; American Heart Association; American Stroke Association Stroke Council; Clinical Cardiology Council; Cardiovascular Radiology and Intervention Council; Atherosclerotic Peripheral Vascular Disease and Quality of Care Outcomes in Research Interdisciplinary Working Groups. Guidelines for the early management of adults with ischemic stroke: a guideline from the American Heart Association/American Stroke Association Stroke Council, Clinical Cardiology Council, Cardiovascular Radiology and Intervention Council, and the Atherosclerotic Peripheral Vascular Disease and Quality of Care Outcomes in Research Interdisciplinary Working Groups: the American Academy of Neurology affirms the value of this guideline as an educational tool for neurologists. Stroke. 2007;38(5):1655-1711. doi:10.1161/STROKEAHA.107.181486 17431204

[17] Zhang D, Wang G, Zhu W, . Expansion of telestroke services improves quality of care provided in super rural areas. Health Aff (Millwood). 2018;37(12):2005-2013. doi:10.1377/hlthaff.2018.05089 30633675

[18] Yu J, Mink PJ, Huckfeldt PJ, Gildemeister S, Abraham JM. Population-level estimates of telemedicine service provision using an all-payer claims database. Health Aff (Millwood). 2018;37(12):1931-1939. doi:10.1377/hlthaff.2018.05116 30633676 PMC13094503

[19] Adeoye O, Nyström KV, Yavagal DR, . Recommendations for the establishment of stroke systems of care: a 2019 update: a policy statement from the American Stroke Association. Stroke. 2019;50(7):e187-e210. doi:10.1161/STR.0000000000000173 31104615

[20] Kepplinger J, Barlinn K, Deckert S, Scheibe M, Bodechtel U, Schmitt J. Safety and efficacy of thrombolysis in telestroke: a systematic review and meta-analysis. Neurology. 2016;87(13):1344-1351. doi:10.1212/WNL.0000000000003148 27566746

[21] Bekelis K, Marth NJ, Wong K, Zhou W, Birkmeyer JD, Skinner J. Primary stroke center hospitalization for elderly stroke patients: implications for case-fatality and travel times. JAMA Intern Med. 2016;176(9):1361-1368. doi:10.1001/jamainternmed.2016.3919 27455403 PMC5434865

[22] Lichtman JH, Allen NB, Wang Y, Watanabe E, Jones SB, Goldstein LB. Stroke patient outcomes in US hospitals before the start of the Joint Commission Primary Stroke Center certification program. Stroke. 2009;40(11):3574-3579. doi:10.1161/STROKEAHA.109.561472 19797179 PMC2782858

[23] Lichtman JH, Jones SB, Wang Y, Watanabe E, Leifheit-Limson E, Goldstein LB. Outcomes after ischemic stroke for hospitals with and without Joint Commission–certified primary stroke centers. Neurology. 2011;76(23):1976-1982. doi:10.1212/WNL.0b013e31821e54f3 21543736 PMC3109877

[24] Lattimore SU, Chalela J, Davis L, ; NINDS Suburban Hospital Stroke Center. Impact of establishing a primary stroke center at a community hospital on the use of thrombolytic therapy: the NINDS Suburban Hospital Stroke Center experience. Stroke. 2003;34(6):e55-e57. doi:10.1161/01.STR.0000073789.12120.F3 12750543

[25] Zachrison KS, Cash RE, Adeoye O, . Estimated population access to acute stroke and telestroke centers in the US, 2019. JAMA Netw Open. 2022;5(2):e2145824. doi:10.1001/jamanetworkopen.2021.45824 35138392 PMC8829668

[26] Shen YC, Chen G, Hsia RY. Community and hospital factors associated with stroke center certification in the United States, 2009 to 2017. JAMA Netw Open. 2019;2(7):e197855. doi:10.1001/jamanetworkopen.2019.7855 31348507 PMC6661722

[27] Hsia RY, Sarkar N, Shen YC. Provision of stroke care services by community disadvantage status in the US, 2009-2022. JAMA Netw Open. 2024;7(7):e2421010. doi:10.1001/jamanetworkopen.2024.21010 39052294 PMC11273237

[28] Shen YC, Sarkar N, Hsia RY. Structural inequities for historically underserved communities in the adoption of stroke certification in the United States. JAMA Neurol. 2022;79(8):777-786. doi:10.1001/jamaneurol.2022.1621 35759253 PMC9237804

[29] US Health Resources & Services Administration. Federal Office of Rural Health Policy (FORHP) data files. Updated February 2025. Accessed July 1, 2021. https://www.hrsa.gov/rural-health/about-us/what-is-rural/data-files

[30] Feldmeier M, Kim AS, Zachrison KS, Alberts MJ, Shen YC, Hsia RY. Heterogeneity of state stroke center certification and designation processes. Stroke. 2024;55(4):1051-1058. doi:10.1161/STROKEAHA.123.045368 38469729 PMC10978226

[31] Kumamaru H, Judd SE, Curtis JR, . Validity of claims-based stroke algorithms in contemporary Medicare data: reasons for geographic and racial differences in stroke (REGARDS) study linked with Medicare claims. Circ Cardiovasc Qual Outcomes. 2014;7(4):611-619. doi:10.1161/CIRCOUTCOMES.113.000743 24963021 PMC4109622

[32] Centers for Medicare and Medicaid Services (CMS), The Joint Commission. Hospital inpatient specifications manuals. Version 5.3a. Accessed October 7, 2024. https://qualitynet.cms.gov/inpatient/specifications-manuals#tab8

[33] Andrade SE, Harrold LR, Tjia J, . A systematic review of validated methods for identifying cerebrovascular accident or transient ischemic attack using administrative data. Pharmacoepidemiol Drug Saf. 2012;21(suppl 1):100-128. doi:10.1002/pds.2312 22262598 PMC3412674

[34] Shen YC, Kim AS, Hsia RY. Treatments and patient outcomes following stroke center expansion. JAMA Netw Open. 2024;7(11):e2444683. doi:10.1001/jamanetworkopen.2024.44683 39535793 PMC11561690

[35] Grigoryan M, Chaudhry SA, Hassan AE, Suri FK, Qureshi AI. Neurointerventional procedural volume per hospital in United States: implications for comprehensive stroke center designation. Stroke. 2012;43(5):1309-1314. doi:10.1161/STROKEAHA.111.636076 22382160

[36] Zachrison KS, Li S, Reeves MJ, . Strategy for reliable identification of ischaemic stroke, thrombolytics and thrombectomy in large administrative databases. Stroke Vasc Neurol. 2021;6(2):194-200. doi:10.1136/svn-2020-000533 33177162 PMC8258073

[37] Specifications manual for Joint Commission national quality measures (v2023B). Accessed January 3, 2024. https://manual.jointcommission.org/releases/TJC2023B/AppendixATJC.html#Table_Number_8.1b:_Mechanical_Endovascular_Reperfusion_Procedures

[38] Powers WJ, Rabinstein AA, Ackerson T, . Guidelines for the early management of patients with acute ischemic stroke: 2019 update to the 2018 guidelines for the early management of acute ischemic stroke: a guideline for healthcare professionals from the American Heart Association/American Stroke Association. Stroke. 2019;50(12):e344-e418. doi:10.1161/STR.0000000000000211 31662037

[39] Saver JL, Goyal M, Bonafe A, ; SWIFT PRIME Investigators. Stent-retriever thrombectomy after intravenous t-PA vs. t-PA alone in stroke. N Engl J Med. 2015;372(24):2285-2295. doi:10.1056/NEJMoa1415061 25882376

[40] Nogueira RG, Jadhav AP, Haussen DC, ; DAWN Trial Investigators. Thrombectomy 6 to 24 hours after stroke with a mismatch between deficit and infarct. N Engl J Med. 2018;378(1):11-21. doi:10.1056/NEJMoa1706442 29129157

[41] Albers GW, Marks MP, Kemp S, ; DEFUSE 3 Investigators. Thrombectomy for stroke at 6 to 16 hours with selection by perfusion imaging. N Engl J Med. 2018;378(8):708-718. doi:10.1056/NEJMoa1713973 29364767 PMC6590673

[42] Wooldridge JM. Econometric Analysis of Cross Section and Panel Data. 2nd ed. MIT Press; 2010.

[43] Shen YC, Hsia RY. Does decreased access to emergency departments affect patient outcomes? analysis of acute myocardial infarction population 1996-2005. Health Serv Res. 2012;47(1 Pt 1):188-210. doi:10.1111/j.1475-6773.2011.01319.x 22091922 PMC3258371

[44] Elixhauser A, Steiner C, Harris DR, Coffey RM. Comorbidity measures for use with administrative data. Med Care. 1998;36(1):8-27. doi:10.1097/00005650-199801000-00004 9431328

[45] Bhattacharya P, Mada F, Salowich-Palm L, . Are racial disparities in stroke care still prevalent in certified stroke centers? J Stroke Cerebrovasc Dis. 2013;22(4):383-388. doi:10.1016/j.jstrokecerebrovasdis.2011.09.018 22078781

[46] Mochari-Greenberger H, Xian Y, Hellkamp AS, . Racial/ethnic and sex differences in emergency medical services transport among hospitalized US stroke patients: analysis of the national Get With The Guidelines-Stroke Registry. J Am Heart Assoc. 2015;4(8):e002099. doi:10.1161/JAHA.115.002099 26268882 PMC4599467

[47] Racial and ethnic disparities in EMS system activation from 2019-2021. JEMS: EMS, Emergency Medical Services—Training, Paramedic, EMT News. May 4, 2023. Accessed October 8, 2024. https://www.jems.com/patient-care/racial-and-ethnic-disparities-in-ems-system-activation-from-2019-2021/

[48] Siegler JE, Boehme AK, Albright KC, Martin-Schild S. Ethnic disparities trump other risk factors in determining delay to emergency department arrival in acute ischemic stroke. Ethn Dis. 2013;23(1):29-34. 23495619 PMC4772663

[49] Boehme AK, Siegler JE, Mullen MT, . Racial and gender differences in stroke severity, outcomes, and treatment in patients with acute ischemic stroke. J Stroke Cerebrovasc Dis. 2014;23(4):e255-e261. doi:10.1016/j.jstrokecerebrovasdis.2013.11.003 24468069 PMC3989836

[50] Karve SJ, Balkrishnan R, Mohammad YM, Levine DA. Racial/ethnic disparities in emergency department waiting time for stroke patients in the United States. J Stroke Cerebrovasc Dis. 2011;20(1):30-40. doi:10.1016/j.jstrokecerebrovasdis.2009.10.006 20538484

[51] Ikeme S, Kottenmeier E, Uzochukwu G, Brinjikji W. Evidence-based disparities in stroke care metrics and outcomes in the United States: a systematic review. Stroke. 2022;53(3):670-679. doi:10.1161/STROKEAHA.121.036263 35105178

[52] Hsia AW, Edwards DF, Morgenstern LB, . Racial disparities in tissue plasminogen activator treatment rate for stroke: a population-based study. Stroke. 2011;42(8):2217-2221. doi:10.1161/STROKEAHA.111.613828 21719765 PMC3148849

[53] Man S, Xian Y, Holmes DN, . Association between thrombolytic door-to-needle time and 1-year mortality and readmission in patients with acute ischemic stroke. JAMA. 2020;323(21):2170-2184. doi:10.1001/jama.2020.5697 32484532 PMC7267850

[54] Muruet W, Rudd A, Wolfe CDA, Douiri A. Long-term survival after intravenous thrombolysis for ischemic stroke: a propensity score-matched cohort with up to 10-year follow-up. Stroke. 2018;49(3):607-613. doi:10.1161/STROKEAHA.117.019889 29440582 PMC5839705

[55] Kwiatkowski TG, Libman RB, Frankel M, ; National Institute of Neurological Disorders and Stroke Recombinant Tissue Plasminogen Activator Stroke Study Group. Effects of tissue plasminogen activator for acute ischemic stroke at one year. N Engl J Med. 1999;340(23):1781-1787. doi:10.1056/NEJM199906103402302 10362821

