# Supplementary material for: Stroke Center Certification and Within-Hospital Racial Disparities in Treatment
Source: JAMA Netw Open. 2025 Jul 30;8(7):e2524027. doi: 10.1001/jamanetworkopen.2025.24027 (PMC12311716; doi:10.1001/jamanetworkopen.2025.24027)
Supplement: Supplement 1. — eMethods. Additional Details on Statistical Models eTable 1. Patient Demographic and Comorbid Characteristics, by Race eTable 2. Complete Regression Results of Figure 2 eTable 3. Changes in Outcomes During the 12- to 24-Month Precertification Period and the Postcertification Period eFigure. Changes in Probabilities of Treatment and Health Outcomes for Black and White Patients With Stroke When Their Admitted Hospital Gains Stroke Certification, Restricted to Hospitals That Treat Patients of Both Races for All Years of the Study Period eReferences. [file jamanetwopen-e2524027-s001.pdf]

Supplementary Online Content

Hsia RY, Shen Y-C. Stroke center certification and within-hospital racial disparities in treatment. *JAMA Netw Open.* 2025;8(7):e2524027.
doi:10.1001/jamanetworkopen.2025.24027

eMethods. Additional Details on Statistical Models
eTable 1. Patient Demographic and Comorbid Characteristics, by Race
eTable 2. Complete Regression Results of Figure 2
eTable 3. Changes in Outcomes During the 12- to 24-Month Precertification Period and the Postcertification Period
eFigure. Changes in Probabilities of Treatment and Health Outcomes for Black and White Patients With Stroke When Their Admitted Hospital Gains Stroke Certification, Restricted to Hospitals That Treat Patients of Both Races for All Years of the Study Period
eReferences.

This supplementary material has been provided by the authors to give readers additional information about their work.

© 2025 Hsia RY and Shen Y-C. *JAMA Network Open*.

eMethods. Additional Details on Statistical Models

Our empirical model followed a difference-in-differences framework, which follows the same principles as a case crossover design. In essence, we compare changes in patient outcomes between pre- and post-periods in the treatment hospital (hospitals that became certified stroke center) to changes in outcomes among patients in the control group (hospital that had no change in stroke center status) according to the following linear probability model (LPM) specification:

$$\mathrm{Y}_{ijt} = \alpha_t + Z_j + \sum_{k=1}^{6} \beta_k (\mathrm{Race}_i \times \mathrm{Cert}_{jt}) + \sum_{l} \beta_l X_{ijt} + \rho_{ijt}$$

Where Y_{ijt} represents the outcome of patient *i* admitted to hospital *j* in year-quarter *t*. The coefficient α_t is a set of year dummies to control for secular trends across all hospitals, and Z_j represents the hospital fixed effects. The hospital fixed effects were critical to our identification strategy because they controlled for systematic unobserved differences across hospitals, including any baseline differences in underlying patient health and socioeconomic conditions. The key coefficients of interest are β_1—β_6, where $Race_i$ is patient *i*'s race (white or black), and $Cert_{jt}$ turns to 1 on and after year-quarter *t* that hospital *j* is certified as stroke centers. There are three categories of certification—ASRH, PSC, and TSC/CSC (due to late introduction and small number of TSC, we group them with CSC since both certification levels have the capacity to perform thrombectomy). Finally, X_{ijt} controls for patient's individual demographic and comorbid conditions, including 5-year age groups, sex, whether the patient is dual eligible for Medicaid, whether this is a recurring stroke episode, and 22 comorbid conditions following prior work.[1,2] The complete list of variables and their corresponding coefficients from our main models are included in eTable 2.

© 2025 Hsia RY and Shen Y-C. *JAMA Network Open*.

The advantage of following this DD framework is that it compares changes in outcomes within each hospital over time, thereby accounting for baseline differences between treatment and control hospitals. We intentionally chose the LPM over the logit models because the LPM can consistently estimate the association between changes in stroke center status and dichotomous outcomes in panel data when there is a large number of fixed effects. In contrast, a logit or other models that rely on maximum likelihood estimation (such as probit or modified Poisson models), while appropriate for cross-sectional data, would result in inconsistent estimates in a panel data setting due to the inclusion of a large number of fixed effects.[3] To address the concern that the LPM produces overly narrow confidence intervals, we estimate heteroskedasticity-robust standard errors that are clustered at the hospital level (i.e., it allows for correlation among patients from the same hospital).[4] Lastly, given that we have multiple outcomes, we report Bonferroni-adjusted p-values and 95% confidence intervals.

eTable 1. Patient Demographic and Comorbid Characteristics, by Race

	**White No. (%)**	**Black No. (%)**
N	1,785,789	323,295
**Patient demographics**		
Female	1,005,147 (56.3)	191,971 (59.4)
Age distribution at time of admission		
65–69 years	239,629 (13.4)	82,243 (25.4)
70–74 years	278,501 (15.6)	67,588 (20.9)
75–79 years	312,450 (17.5)	60,192 (18.6)
80–84 years	346,407 (19.4)	50,320 (15.6)
85+ years	608,802 (34.1)	62,952 (19.5)
Medicaid eligible	169,108 (9.5)	89,950 (27.8)
**Patient clinical conditions**		
Recurring stroke	148,525 (8.3)	36,856 (11.4)
Transfer	91,435 (5.1)	11,961 (3.7)
Peripheral vascular disease	182,776 (10.2)	30,434 (9.4)
Pulmonary circulation disorders	63,264 (3.5)	12,472 (3.9)
Diabetes	528,898 (29.6)	144,846 (44.8)

© 2025 Hsia RY and Shen Y-C. *JAMA Network Open*.

Kidney failure	313,883 (17.6)	85,402 (26.4)
Liver	16,934 (0.9)	3,971 (1.2)
Cancer	72,969 (4.1)	13,277 (4.1)
Dementia	190,040 (10.6)	39,653 (12.3)
Valvular disease	186,087 (10.4)	23,104 (7.1)
Hypertension	1,497,140 (83.8)	294,013 (91.0)
Chronic pulmonary disease	290,415 (16.3)	44,817 (13.9)
Rheumatoid arthritis/collagen vascular	52,033 (2.9)	7,160 (2.2)
Coagulation deficiency	67,499 (3.8)	12,984 (4.0)
Obesity	139,563 (7.8)	34,552 (10.7)
Substance use	36,623 (2.1)	10,676 (3.3)
Depression	186,913 (10.5)	18,818 (5.8)
Psychosis	128,612 (7.2)	16,540 (5.1)
Hypothyroidism	344,380 (19.3)	27,017 (8.4)
Paralysis and other neurological disorder	1,019,345 (57.1)	190,175 (58.8)
Ulcer	5,847 (0.3)	1,193 (0.4)
Weight loss	74,349 (4.2)	19,366 (6.0)
Fluid and electrolyte disorders	398,284 (22.3)	85,745 (26.5)
Anemia (blood loss and deficiency)	209,148 (11.7)	59,330 (18.4)

© 2025 Hsia RY and Shen Y-C. *JAMA Network Open*.

eTable 2. Complete Regression Results of Figure 2

	**Received thrombolytic therapy**	**Received thrombectomy**	**Home at 90 days**	**1-year mortality**
On and after year-quarter gaining ASRH certification				
White	0.58	-0.53	-0.09	-0.35
	[-0.82,1.99]	[-1.79,0.72]	[-1.15,0.97]	[-1.37,0.66]
Black	-0.83	-0.93	0.63	0.92
	[-2.12,0.46]	[-2.51,0.65]	[-1.00,2.27]	[-0.97,2.81]
On and after year-quarter gaining PSC certification				
White	1.70**	-0.68**	0.25	-0.16
	[1.19,2.21]	[-1.00,-0.36]	[-0.20,0.71]	[-0.59,0.26]
Black	0.07	-1.11**	-0.03	0.13
	[-0.53,0.68]	[-1.49,-0.74]	[-0.86,0.80]	[-0.60,0.86]
On and after year-quarter gaining TSC or CSC certification				
White	3.76**	3.74**	-0.73*	0.42
	[2.89,4.62]	[3.02,4.45]	[-1.44,-0.02]	[-0.23,1.07]
Black	0.02	0.97*	0.21	0.64
	[-1.30,1.34]	[-0.00,1.93]	[-0.96,1.38]	[-0.33,1.60]
**Patient characteristics**				
Black	-1.15**	-0.37**	0.61	-1.08**
	[-1.50,-0.81]	[-0.55,-0.19]	[-0.08,1.29]	[-1.69,-0.47]
Female	-0.24**	0.26**	-1.93**	0.38**
	[-0.36,-0.12]	[0.19,0.32]	[-2.10,-1.76]	[0.22,0.55]
Medicaid eligible	-1.33**	-0.63**	0.63**	-9.84**
	[-1.51,-1.15]	[-0.75,-0.51]	[0.19,1.07]	[-10.14,-9.54]
Age at time of admission	-0.09**	-0.04**	-1.02**	1.19**
	[-0.09,-0.08]	[-0.05,-0.04]	[-1.03,-1.00]	[1.18,1.21]
Age squared	-0.00**	-0.00**	-0.04**	0.06**
	[-0.00,-0.00]	[-0.00,-0.00]	[-0.04,-0.04]	[0.05,0.06]
**Clinical characteristics**				
Recurrent stroke	-2.14**	-0.72**	-4.61**	5.41**
	[-2.35,-1.93]	[-0.85,-0.60]	[-4.91,-4.32]	[5.13,5.69]
Peripheral vascular disease	-0.66**	0.37**	-1.08**	2.24**
	[-0.85,-0.47]	[0.25,0.49]	[-1.38,-0.79]	[1.95,2.53]
Pulmonary circulation disorders	1.03**	0.94**	-6.54**	8.14**
	[0.72,1.34]	[0.72,1.15]	[-7.03,-6.05]	[7.66,8.63]
Diabetes	-1.57**	-0.55**	-2.56**	2.70**
	[-1.70,-1.45]	[-0.63,-0.47]	[-2.74,-2.38]	[2.53,2.86]
Kidney failure	-1.26**	-0.65**	-5.12**	8.19**
	[-1.43,-1.09]	[-0.76,-0.55]	[-5.35,-4.88]	[7.96,8.43]
Liver disease	-2.52**	-0.82**	-4.35**	7.09**
	[-3.04,-2.00]	[-1.13,-0.52]	[-5.17,-3.54]	[6.31,7.86]
Cancer	-3.46**	-0.52**	-19.66**	30.91**
	[-3.75,-3.16]	[-0.70,-0.34]	[-20.14,-19.19]	[30.41,31.41]

© 2025 Hsia RY and Shen Y-C. *JAMA Network Open*.

Dementia	-3.17**	-1.63**	-10.77**	11.97**
	[-3.38,-2.96]	[-1.81,-1.46]	[-11.10,-10.45]	[11.65,12.30]
Valvular disease	0.05	0.09	2.03**	-0.36**
	[-0.14,0.24]	[-0.02,0.21]	[1.75,2.30]	[-0.65,-0.07]
Hypertension	0.03	-0.27**	5.80**	-6.44**
	[-0.14,0.19]	[-0.38,-0.17]	[5.54,6.05]	[-6.69,-6.20]
Chronic pulmonary disease	0.09	-0.10**	-3.12**	5.78**
	[-0.06,0.24]	[-0.18,-0.02]	[-3.34,-2.90]	[5.56,6.01]
Rheumatoid arthritis/collagen vascular disorders	-0.35*	-0.47**	0.91**	0.57**
	[-0.67,-0.03]	[-0.63,-0.31]	[0.44,1.37]	[0.11,1.04]
Coagulation deficiency	0.75**	1.86**	-6.45**	7.17**
	[0.38,1.12]	[1.58,2.15]	[-6.96,-5.94]	[6.70,7.65]
Obesity	0.54**	0.48**	0.73**	-2.88**
	[0.30,0.78]	[0.32,0.63]	[0.45,1.02]	[-3.15,-2.61]
Substance use	-1.41**	-0.47**	-1.52**	-0.36
	[-1.81,-1.02]	[-0.70,-0.24]	[-2.07,-0.96]	[-0.86,0.14]
Depression	-0.98**	-0.50**	-0.39*	-0.50**
	[-1.19,-0.77]	[-0.59,-0.40]	[-0.74,-0.03]	[-0.82,-0.17]
Psychosis	-0.41**	-0.81**	-2.00**	1.00**
	[-0.66,-0.16]	[-0.96,-0.67]	[-2.42,-1.58]	[0.62,1.37]
Hypothyroidism	0.06	-0.01	1.53**	-1.40**
	[-0.08,0.21]	[-0.09,0.07]	[1.32,1.75]	[-1.61,-1.19]
Paralysis and other neurological disorders	8.89**	2.75**	-13.78**	9.93**
	[8.57,9.20]	[2.48,3.02]	[-14.01,-13.54]	[9.74,10.12]
Chronic peptic ulcer disease	-0.07	2.47**	-8.62**	5.12**
	[-1.16,1.01]	[1.56,3.37]	[-10.17,-7.07]	[3.70,6.54]
Weight loss	-0.82**	0.79**	-21.78**	21.12**
	[-1.24,-0.39]	[0.47,1.12]	[-22.32,-21.25]	[20.61,21.62]
Fluid and electrolyte disorders	-0.35**	1.15**	-12.01**	10.07**
	[-0.56,-0.14]	[0.99,1.31]	[-12.33,-11.70]	[9.79,10.34]
Anemia (blood loss and deficiency)	1.03**	1.22**	-4.64**	6.02**
	[0.81,1.26]	[1.02,1.41]	[-4.94,-4.35]	[5.73,6.31]
On and after ICD-10 switch (Oct 2015)	0.94**	-0.25	3.47**	-6.43**
	[0.35,1.54]	[-0.56,0.05]	[2.68,4.26]	[-7.17,-5.70]
Admitted in 2010	0.67**	0.14**	1.14**	-0.64**
	[0.44,0.90]	[0.04,0.23]	[0.76,1.53]	[-1.01,-0.27]
Admitted in 2011	1.37**	0.09	3.57**	-3.11**
	[1.08,1.66]	[-0.04,0.22]	[3.17,3.97]	[-3.49,-2.73]
Admitted in 2012	2.03**	0.18**	4.34**	-3.08**
	[1.70,2.37]	[0.03,0.33]	[3.91,4.77]	[-3.48,-2.68]
Admitted in 2013	3.10**	0.18*	5.22**	-3.95**
	[2.75,3.46]	[0.00,0.35]	[4.79,5.65]	[-4.35,-3.56]
Admitted in 2014	3.70**	0.22*	5.83**	-4.18**
	[3.32,4.07]	[0.02,0.42]	[5.40,6.25]	[-4.57,-3.79]

© 2025 Hsia RY and Shen Y-C. *JAMA Network Open*.

Admitted in 2015	4.33**	0.92**	7.10**	-4.88**
	[3.90,4.75]	[0.67,1.16]	[6.64,7.56]	[-5.32,-4.43]
Admitted in 2016	4.30**	1.54**	7.22**	-1.01**
	[3.59,5.02]	[1.15,1.94]	[6.33,8.12]	[-1.86,-0.17]
Admitted in 2017	4.85**	2.30**	8.16**	-1.32**
	[4.13,5.58]	[1.85,2.75]	[7.24,9.08]	[-2.17,-0.46]
Admitted in 2018	5.26**	3.36**	9.24**	-2.18**
	[4.53,6.00]	[2.87,3.85]	[8.32,10.16]	[-3.04,-1.31]
Admitted in 2019	5.12**	3.84**	10.95**	-2.61**
	[4.39,5.85]	[3.32,4.35]	[10.04,11.86]	[-3.48,-1.73]
constant	2.16**	0.47**	78.68**	20.17**
	[1.71,2.61]	[0.18,0.75]	[78.16,79.20]	[19.69,20.65]
N	2,109,075	2,109,075	2,109,075	2,109,075

Note: *<0.05 **<0.01. p-value and 95% confidence intervals are Bonferroni adjusted to account for multiple testing. Abbreviations: ASRH, Acute Stroke Ready Hospital; PSC, Primary Stroke Center; TSC, Thrombectomy Capable Stroke Center; CSC, Comprehensive Stroke Center. Error bars represent 95% confidence intervals.

© 2025 Hsia RY and Shen Y-C. *JAMA Network Open*.

eTable 3. Changes in Outcomes During the 12- to 24-Month Precertification Period and the Postcertification Period

	Received thrombolytic therapy	Received thrombectomy	Home at 90 days	1-year mortality
**White patients**				
**ASRH**				
12-24 months prior	-0.63	-0.64	3.77	-6.17**
	[-3.04,1.78]	[-1.56,0.29]	[-0.20,7.74]	[-10.26,-2.08]
on and after certified	0.62	-0.47	-0.01	-0.45
	[-0.80,2.04]	[-1.75,0.81]	[-1.07,1.06]	[-1.47,0.57]
**PSC**				
12-24 months prior	0.43	-0.32*	0.97	-0.30
	[-0.50,1.37]	[-0.60,-0.04]	[-0.41,2.35]	[-1.63,1.03]
on and after certified	1.75**	-0.65**	0.31	-0.19
	[1.23,2.26]	[-0.97,-0.33]	[-0.15,0.78]	[-0.62,0.24]
**TSC or CSC**				
12-24 months prior	1.54**	2.43**	0.35	-0.14
	[0.75,2.33]	[1.81,3.04]	[-0.49,1.19]	[-0.86,0.59]
on and after certified	3.97**	4.04**	-0.63	0.38
	[3.06,4.87]	[3.30,4.78]	[-1.37,0.12]	[-0.30,1.06]
**Black patients**				
**ASRH**				
12-24 months prior	-0.77	-0.26	0.65	2.28
	[-7.58,6.05]	[-1.61,1.09]	[-12.51,13.81]	[-5.12,9.68]
on and after certified	-0.77	-0.86	0.70	0.86
	[-2.06,0.52]	[-2.46,0.75]	[-0.93,2.34]	[-1.03,2.74]
**PSC**				
12-24 months prior	1.05	-0.05	1.13	0.22
	[-0.72,2.81]	[-0.46,0.37]	[-2.65,4.92]	[-3.46,3.90]
on and after certified	0.15	-1.05**	0.02	0.13
	[-0.46,0.76]	[-1.43,-0.68]	[-0.82,0.86]	[-0.59,0.85]
**TSC or CSC**				
12-24 months prior	0.07	0.95*	0.37	-0.05
	[-1.29,1.44]	[0.02,1.87]	[-1.06,1.80]	[-1.57,1.48]
on and after certified	0.21	1.25**	0.31	0.62
	[-1.14,1.56]	[0.27,2.24]	[-0.89,1.50]	[-0.36,1.60]
N	2,109,075	2,109,075	2,109,075	2,109,075

Note: *<0.05 **<0.01. p-value and 95% confidence intervals are Bonferroni adjusted to account for multiple testing. Abbreviations: ASRH, Acute Stroke Ready Hospital; PSC, Primary Stroke Center; TSC, Thrombectomy Capable Stroke Center; CSC, Comprehensive Stroke Center. Error bars represent 95% confidence intervals.

© 2025 Hsia RY and Shen Y-C. *JAMA Network Open*.

eFigure. Changes in Probabilities of Treatment and Health Outcomes for Black and White Patients With Stroke When Their Admitted Hospital Gains Stroke Certification, Restricted to Hospitals That Treat Patients of Both Races for All Years of the Study Period

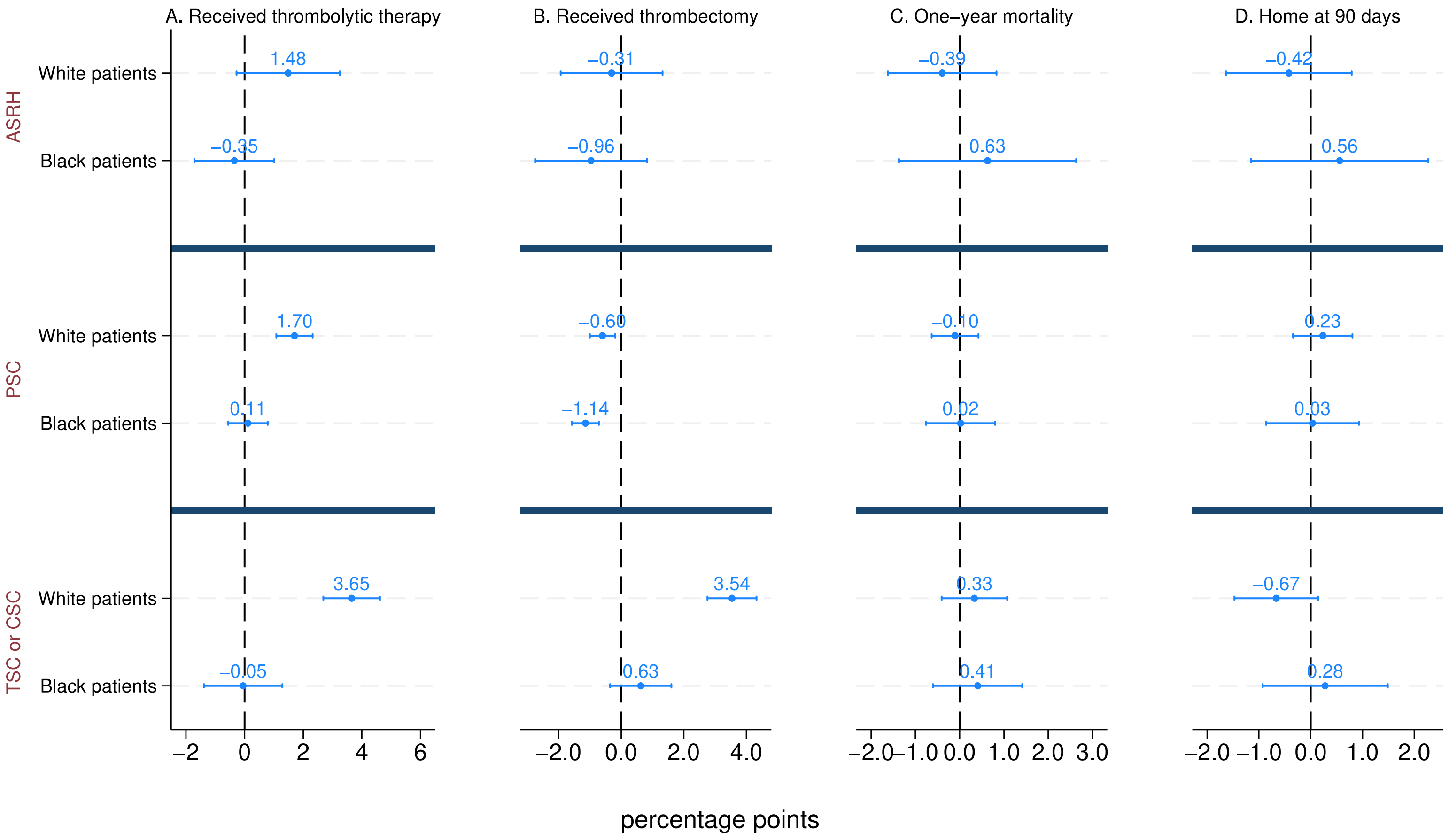

Note: N= 1,604,801. Error bars represent 95% Bonferroni-adjusted confidence intervals. Abbreviations: ASRH, Acute Stroke Ready Hospital; PSC, Primary Stroke Center; TSC, Thrombectomy Capable Stroke Center; CSC, Comprehensive Stroke Center.

© 2025 Hsia RY and Shen Y-C. *JAMA Network Open*.

eReferences.

1. Shen YC, Hsia RY. Does decreased access to emergency departments affect patient outcomes? Analysis of acute myocardial infarction population 1996-2005. *Health Serv Res*. 2012;47:188-210. doi:10.1111/j.1475-6773.2011.01319.x

2. Elixhauser A, Steiner C, Harris DR, Coffey RM. Comorbidity measures for use with administrative data. *Med Care*. 1998;36(1):8-27. doi:10.1097/00005650-199801000-00004

3. Wooldridge JM. *Econometric Analysis of Cross Section and Panel Data*. 2nd ed. MIT Press; 2010.

4. Stock JH, Watson MW. Heteroskedasticity-Robust Standard Errors for Fixed Effects Panel Data Regression. *Econometrica*. 2008;76(1):155-174. doi:10.1111/j.0012-9682.2008.00821.x

© 2025 Hsia RY and Shen Y-C. *JAMA Network Open*.
