# Supplementary material for: Stroke Center Certification and Within-Hospital Racial Disparities in Treatment
Source: JAMA Netw Open. 2025 Jul 30;8(7):e2524027. doi: 10.1001/jamanetworkopen.2025.24027 (PMC12311716; doi:10.1001/jamanetworkopen.2025.24027)
Supplement: Supplement 2. — Data Sharing Statement [file jamanetwopen-e2524027-s002.pdf]

Data Sharing Statement

Hsia. Stroke Center Certification and Within-Hospital Racial Disparities in Treatment. *JAMA Netw Open*. Published July 30, 2025. doi:10.1001/jamanetworkopen.2025.24027

Data
Data available: No

Additional Information
Explanation for why data not available: Patient-level data from the 100% Medicare Provider and Analysis Review (MedPAR) are not publicly available. Data requests can be sent to https://www.cms.gov/.
